# Supplementary material for: Neuronal lysosomal dysfunction releases exosomes harboring APP C-terminal fragments and unique lipid signatures
Source: Nat Commun. 2018 Jan 18;9:291. doi: 10.1038/s41467-017-02533-w (PMC5773483; doi:10.1038/s41467-017-02533-w)
Supplement: Supplementary file 1 — Supplementary Information [file 41467_2017_2533_MOESM1_ESM.docx]

**
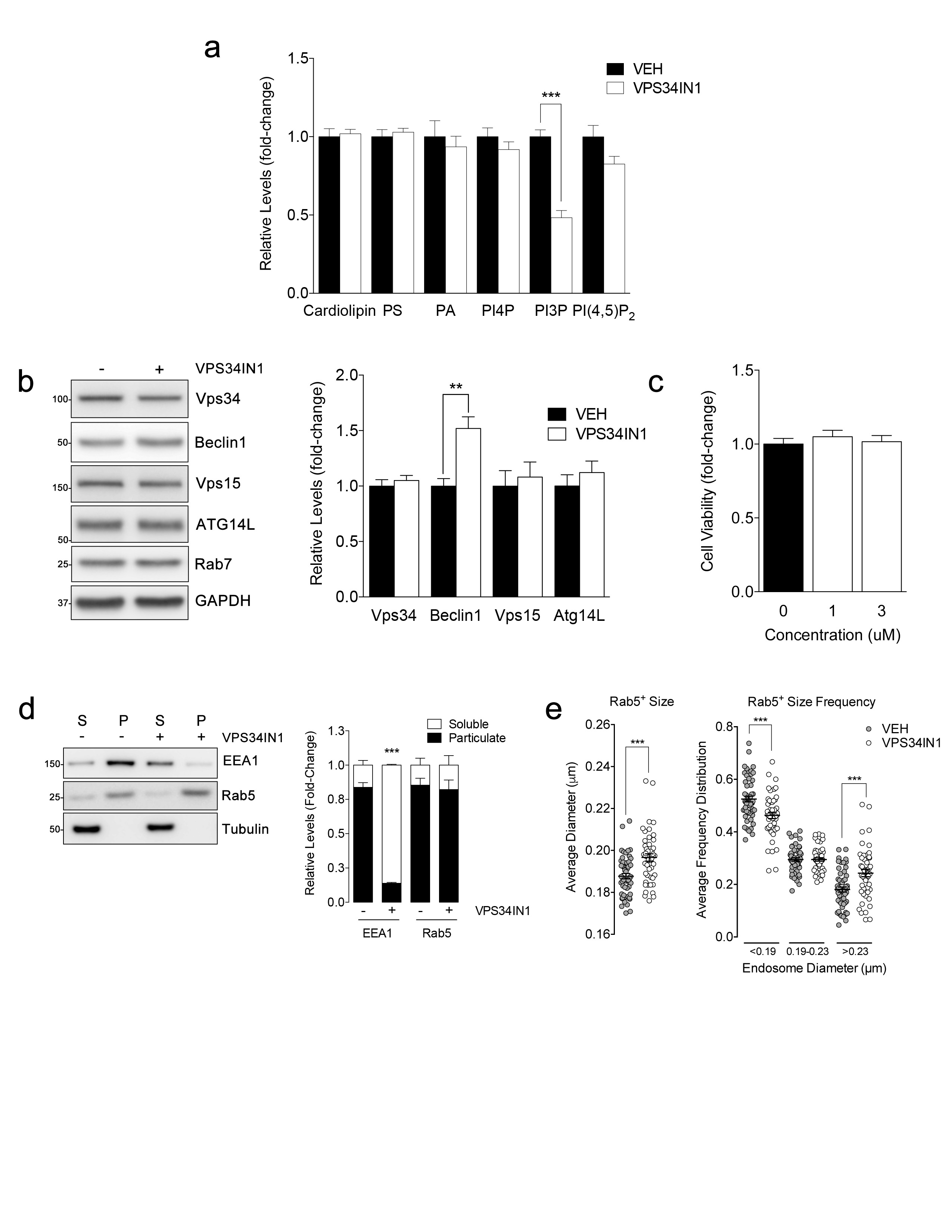
**

**Supplementary Figure 1. VPS34IN1 reduces PI3P production and alters early endosome dynamics. a)** Bar diagram showing lipid levels in N2a cells treated with VPS34IN1 at 1µM for 24 hr. Measurements were made by anionic exchange HPLC with suppressed conductivity detection, expressed in Mol% of total anionic phospholipids measured and normalized to vehicle (mean ± SEM, N=8, from two independent experiments). Phosphatidylserine (PS), Phosphatidic Acid (PA), Phosphatidylinositol-4-phosphate (PI4P), Phosphatidylinositol-3-phosphate (PI3P), Phosphatidylinositol-4,5-bisphosphate (PI(4,5)P_2_). ***p<0.001 in two-tailed Student’s t-test. **b)** Western blot analysis of protein levels of Vps34 interactors in cortical neurons treated with VPS34IN1 at 3µM for 24 hr. Rab7 was used as control. (mean ± SEM, N=4, from two independent experiments). **p<0.01 in two-tailed Student’s t-test. **c)** Cortical neuron cell viability measured after treatment with VPS34IN1 at indicated doses for 24 hr (mean ± SEM, N=20, from two independent experiments). **d)** Western blot analysis of endosomal protein levels of N2a cells treated with VPS34IN1 at 3µM for 3 hr. Cell extracts were subjected to lysis and ultracentrifugation for extraction of soluble (S) or particulate (P) fractions. Values were expressed as fraction of total protein levels (sum of S and P fractions). Tubulin was used as loading control (mean ± SEM, N=3). ***p<0.001 in two-tailed Student’s t-test. **e)** Size analysis of Rab5 puncta from cortical neurons treated with vehicle or VPS34IN1 at 3µM for 3 hr (Figure 1a). Bar graphs indicate average Rab5 puncta size, per cell. Puncta were categorized in three size classes for analysis of relative frequency (mean ± SEM, N=55 and 49, resp.). ***p<0.001 in two-tailed Student’s t-test.

**
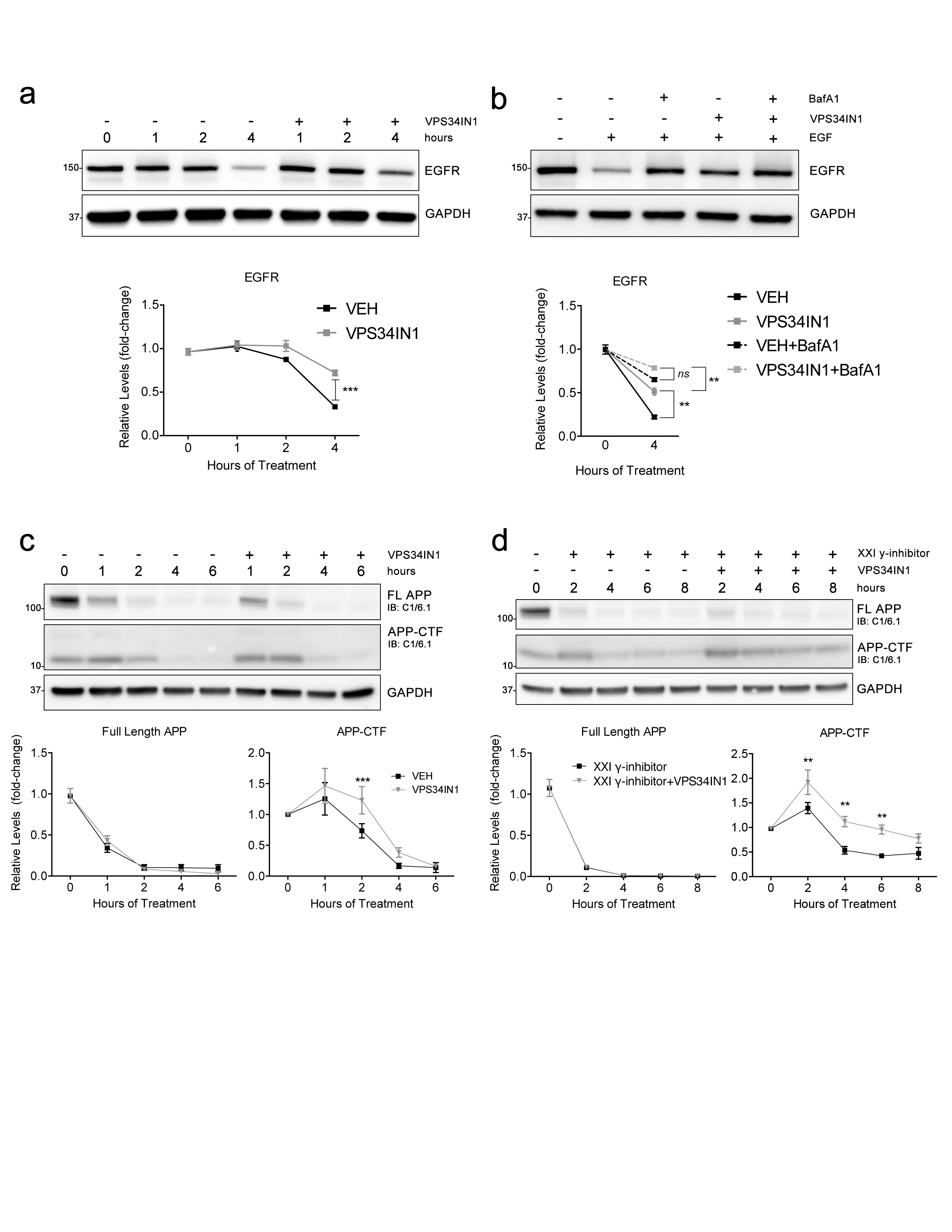
**

**Supplementary Figure 2. VPS34IN1 delays ligand-induced receptor degradation and APP-CTF lysosomal clearance. a)** Western Blot analysis of EGFR levels in cultured cortical neurons after EGF stimulation at 200ng/ml and vehicle or VPS34IN1 treatment at 3µM for the indicated time periods (mean ± SEM, N=3). ***p<0.001 in two-way ANOVA repeated measures, Holm-Sidak’s multiple comparisons test. **b)** Western Blot analysis of EGFR levels in cultured cortical neurons after treatment as in **a)** plus BafA1 at 50nM for 4 hr. (mean ± SEM, N=3). **p<0.01 in two-way ANOVA repeated measures, Holm-Sidak’s multiple comparisons test. **c)** Western Blot analysis of FL-APP and APP-CTF levels in N2a cells after pulse treatment with cycloheximide at 50µg/ml plus vehicle or VPS34IN1 at 1µM for the indicated time periods (mean ± SEM, N=4). ***p<0.001 in two-way ANOVA repeated measures, Holm-Sidak’s multiple comparisons test. **d)** Western Blot analysis of FL-APP and APP-CTF levels in N2a cells treated as in **c)** plus XXI γ–inhibitor at 2µM for the indicated time periods (mean ± SEM, N=4). **p<0.01 in two-way ANOVA repeated measures, Holm-Sidak’s multiple comparisons test.

**
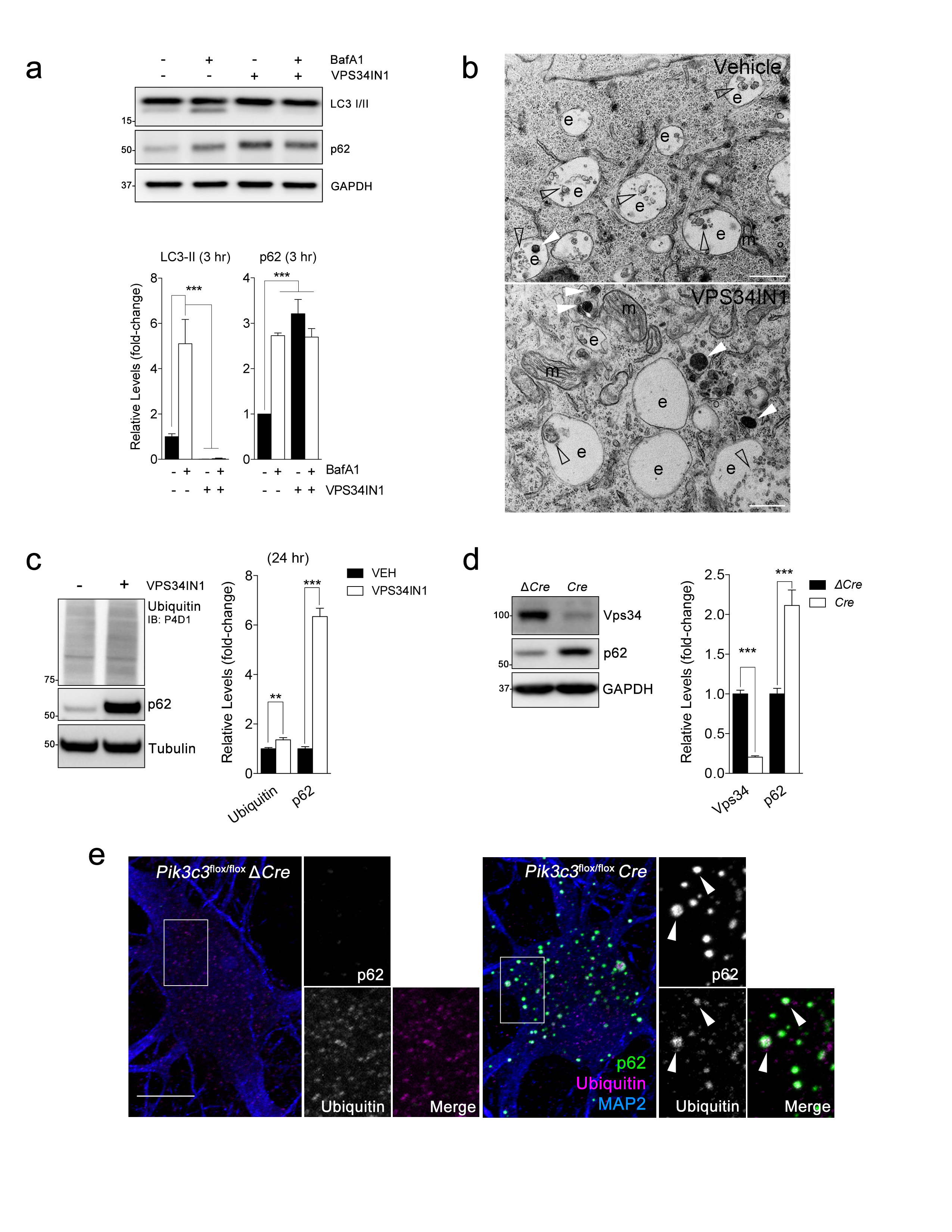
**

**Supplementary Figure 3. VPS34 inhibition blocks autophagosome initiation and causes accumulation of p62 and poly-ubiquitinated proteins.** **a)** Western Blot analysis of LC3 lipidation and p62 protein levels in cortical neurons after treatment with vehicle, Bafilomycin A1 (BafA1) at 50nM, VPS34IN1 at 3µM or co-treatment for 3 hr. Bar graphs denote average protein levels normalized to vehicle. (mean ± SEM, N=6 for LC3, N=3 for p62, from two independent experiments). ***p<0.001 in one-way ANOVA, Holm-Sidak’s multiple comparisons test. **b)** Ultrastructural electron microscopic analysis of endocytic compartments of primary cortical neurons treated with vehicle or VPS34IN1 for 24 hr. Full arrows, electron-dense vesicles; empty arrows, intraluminal vesicles; e, endosome; m, mitochondrion. Scale bar, 500nm. **c)** Western Blot analysis of ubiquitin and p62 levels in cortical neurons after treatment with VPS34IN1 at 3µM for 24 hr. Bar graph indicates average protein levels normalized to vehicle (mean ± SEM, n=8, from two independent experiments). **p<0.01, ***p<0.001 in two-tailed Student’s t-test. **d)** Western blot analysis of Vps34 and p62 levels in *Pik3c3^flox/flox^* cortical neurons virally transduced with inactive (Δ*Cre)* and active *Cre* (*Cre*) recombinase for 7 days. Bar graph indicates average protein levels normalized to Δ*Cre* (mean ± SEM, N=8, from two independent experiments). ***p<0.001 in two-tailed Student’s t-test. **e)** Representative confocal images of *Pik3c3^flox/flox^* cortical neurons virally transduced with Δ*Cre* and *Cre* for 7 days. Arrows highlight p62 and ubiquitin colocalization. Scale bar, 10µm.

**
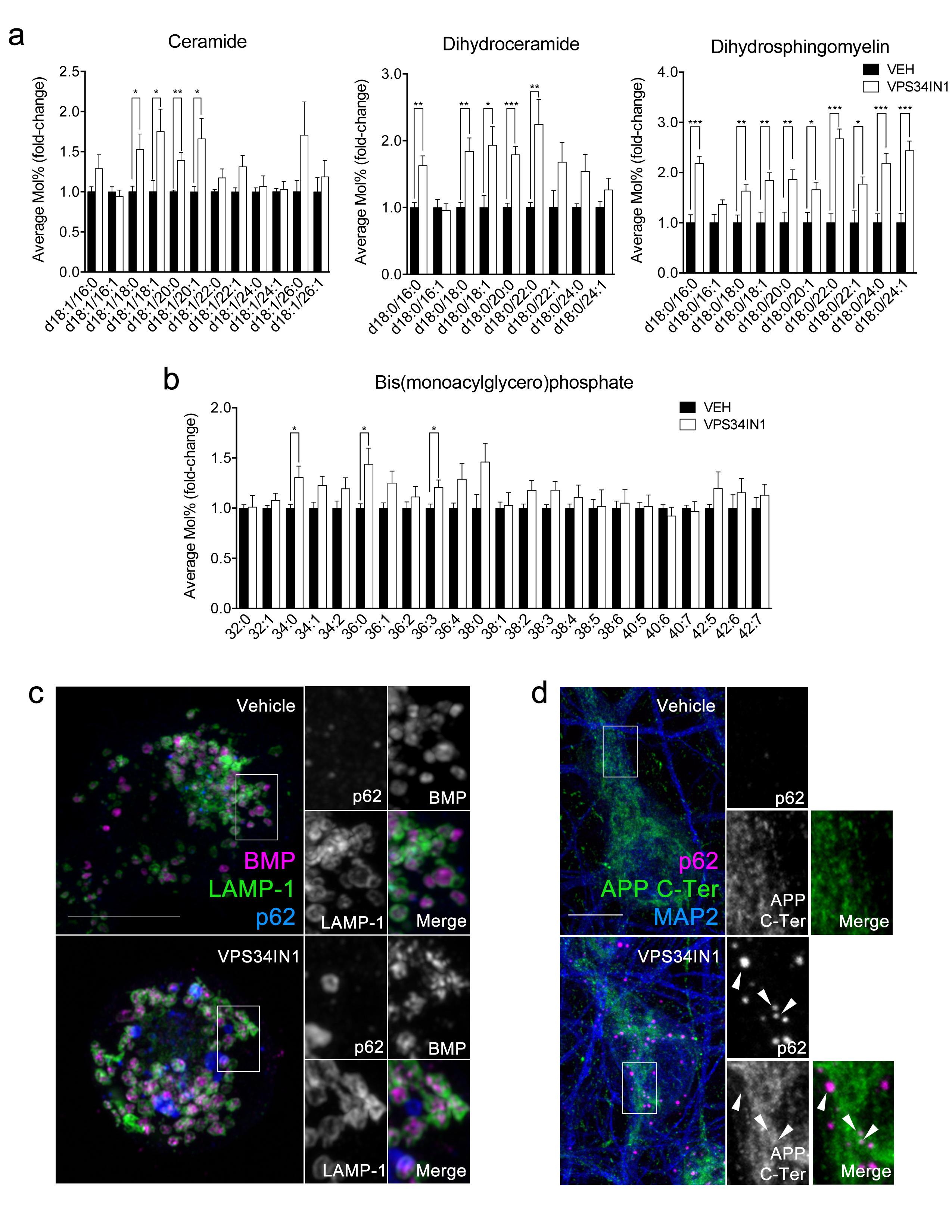
**

**Supplementary Figure 4.** **Effect of VPS34IN1 treatment on lipid metabolism, BMP and APP localization.** **a-b)** Molecular species analysis of ceramide (Cer), dihydroceramide (dhCer), dihydrosphingomyelin (dhSM) and Bis(Monoacylglycero)Phosphate (BMP) from primary cortical neurons treated with vehicle or VPS34IN1 at 3µM for 24 hr. Values were expressed as average Mol% of total lipid measured, normalized to vehicle. Lipids are annotated per total acyl carbons and degree of unsaturation (mean ± SEM, N=8, from two independent experiments) * p<0.05, **p<0.01, ***p<0.001 in two-tailed Student’s t-test. **c)** Representative confocal images of N2a cells treated with vehicle or VPS34IN1 at 1uM for 24hr and immunostained for LAMP-1, p62 and BMP (see Methods). Airyscan insets highlight luminal enrichment of BMP in LAMP-1-positive compartments in both experimental conditions. Scale bar, 10µm. **d)** Representative confocal images of cortical neurons treated with vehicle or VPS34IN1 at 3µM for 24 hr and immunostained for MAP2, APP cytodomain (C1/6.1) and p62. APP C1/6.1 antibody cross-reacts with both FL-APP and APP-CTFs. Arrows highlight p62-positive structures in proximity to APP signal. Scale bar, 10µm.


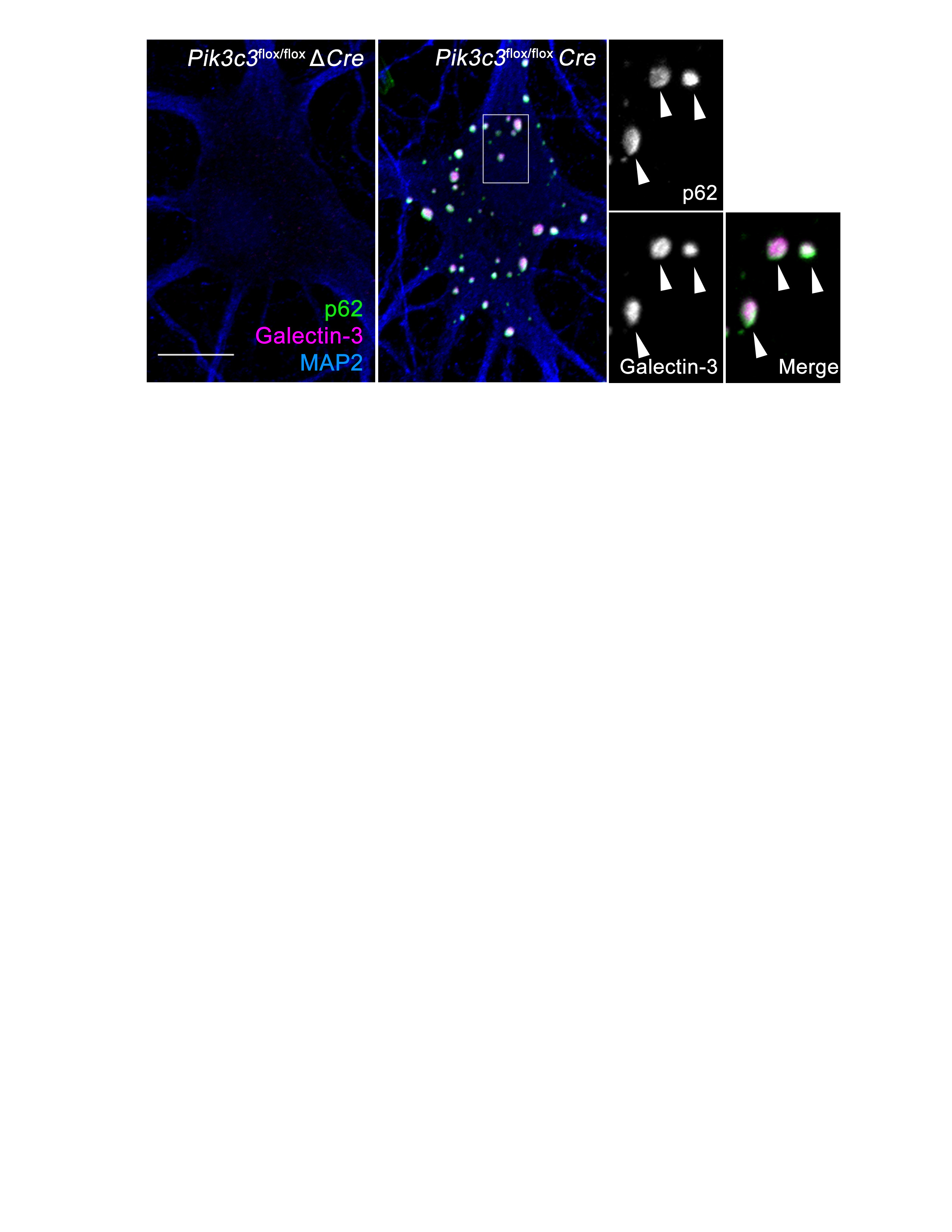


**Supplementary Figure 5. Vps34 KO neurons phenocopy endolysosomal membrane damage detected with Vps34 pharmacological inhibition.** Representative confocal images of cultured cortical neurons 8 days post lentiviral infection with Δ*Cre* or *Cre* recombinase. Arrows highlight p62 and galectin-3 colocalization. Scale bar, 10µm.

**
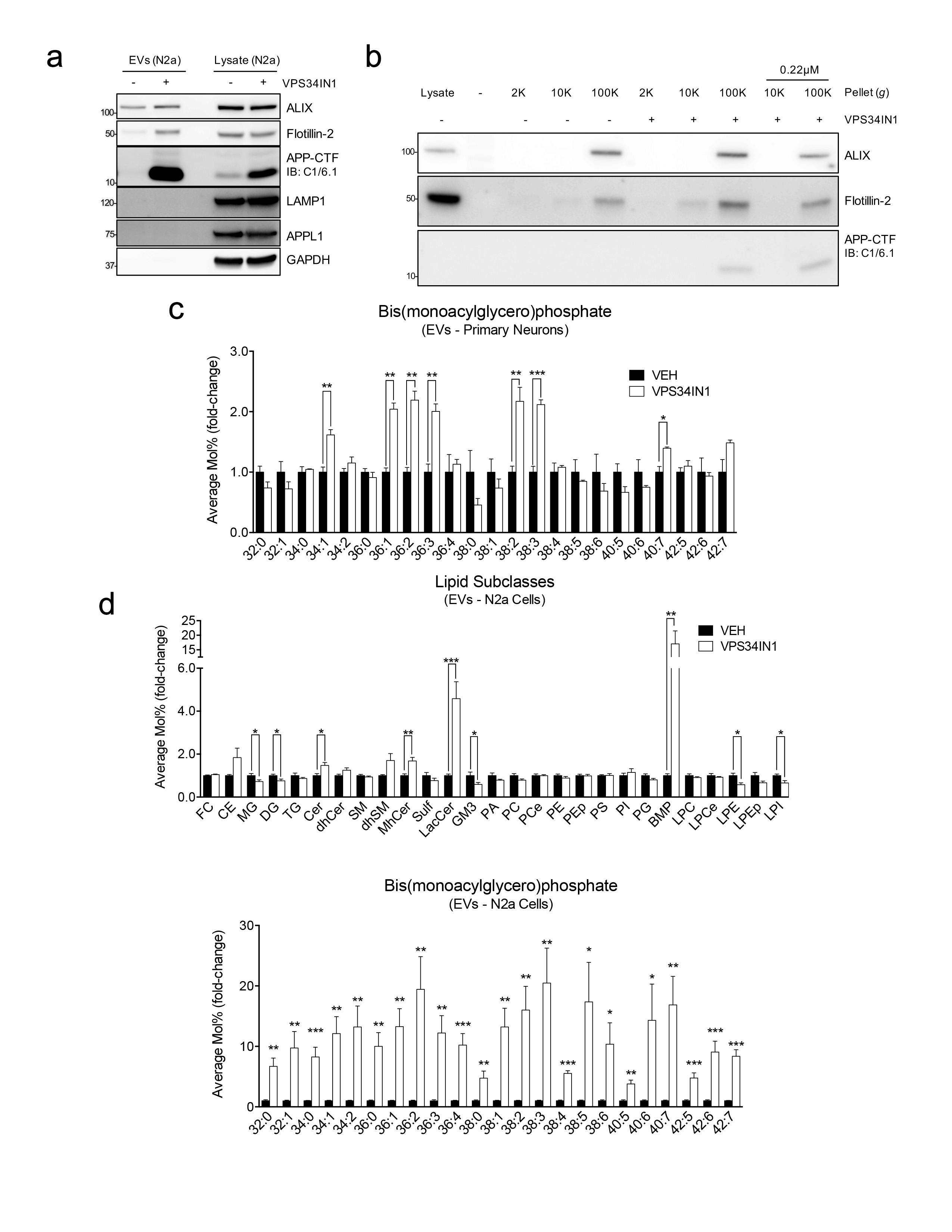
**

**Supplementary Figure 6. EVs secreted upon VPS34IN1 treatment are enriched for multiple BMP species, both in neurons and N2a cells.** **a-b)** Western blot analysis of N2a cell lysates and EV fractions after treatment with vehicle or VPS34IN1 at 1µM for 24hr. Note exclusion of late endosome/lysosomal marker LAMP-1, early endosome marker APPL1 and cytosolic protein GAPDH from EV preparations. **b)** Cell media was sequentially ultracentrifuged at indicated *g*’s or alternatively filtered with a 0.2µm filter and ultracentrifuged at 100,000*g*. EV markers were enriched and APP-CTFs exclusively present in the *100,000g* pellet. **c)** Molecular species analysis of BMP extracted from EVs purified from cortical neuron culture media after treatment with vehicle or VPS34IN1 at 3µM for 24 hr. Values were expressed as average Mol% of total lipid measured, normalized to vehicle. Lipids are annotated per total acyl carbons and degree of unsaturation (mean ± SEM, N=3 each from a pool of two biological replicates) **d)** Upper panel, LC-MS analysis of lipids extracted from EVs collected from N2a cell culture media after treatment with vehicle or VPS34IN1 at 1µM for 24 hr. Lower panel, molecular species analysis of BMP from EVs collected from N2a cell culture media under the same treatment conditions. Values were expressed as average Mol% of total lipids measured, normalized to vehicle (mean ± SEM N=7 each from a pool of two biological replicates) * p<0.05, ** p<0.01, ***P<0.01 in two-tailed Student’s t-test.

**
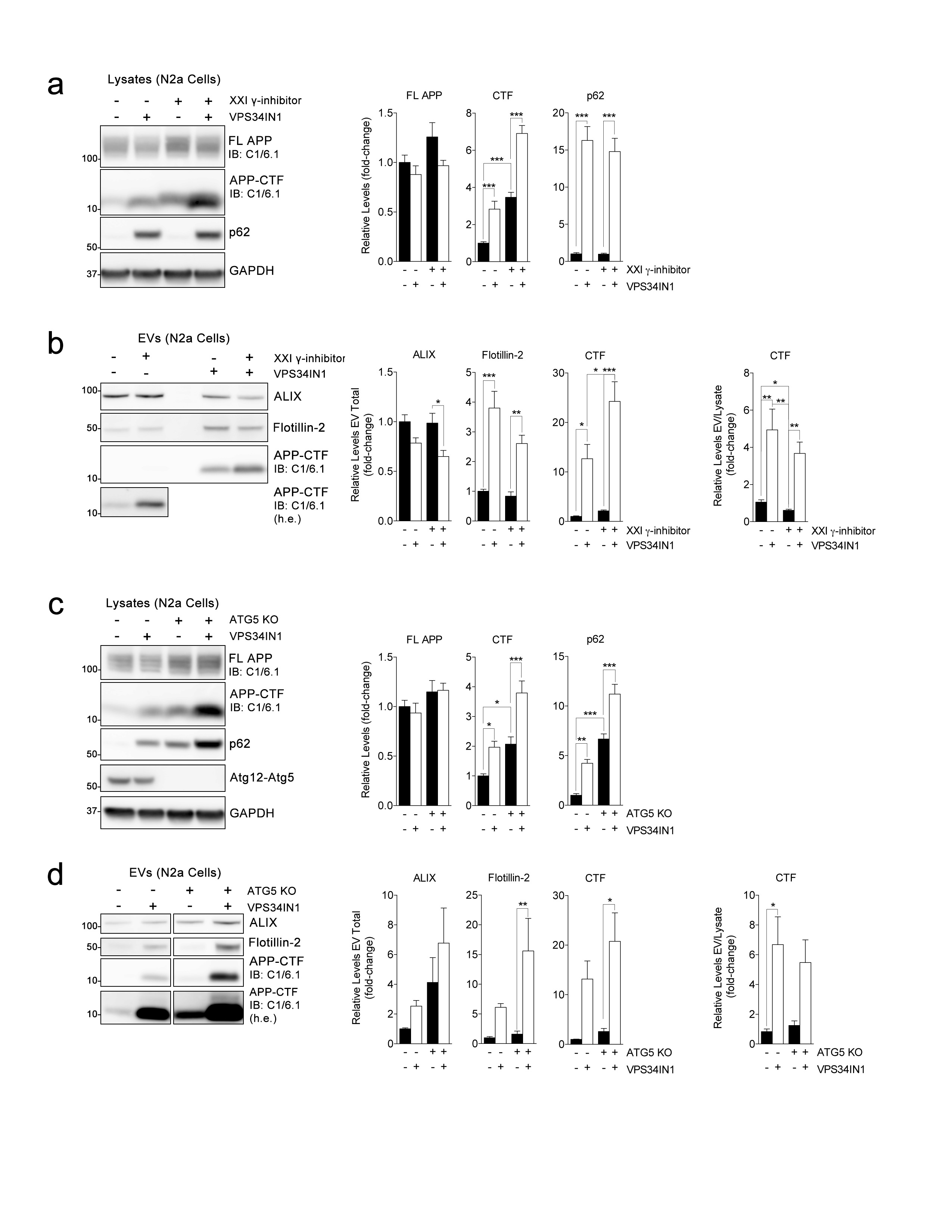
**

**Supplementary Figure 7. Secretion of exosomal APP-CTFs does not simply reflect cellular accumulation of APP-CTFs.** **a-b)** Western blot analysis of cell lysates and EVs from N2a cells treated with vehicle, XXI γ-inhibitor at 2µM, VPS34IN1 at 1µM or co-treated for 24 hr. EV protein levels were normalized to lysate total protein (EV total) or lysate levels of the corresponding protein (EV/Lysate ratio). Bar graph denotes average protein levels normalized to vehicle (mean ± SEM, N=5-6, from two independent experiments). * p<0.05, **p<0.01, ***p<0.001 in one-way ANOVA, Holm-Sidak’s multiple comparisons test. **c-d)** Western blot analysis of cell lysates and EVs from ATG5-KO N2a and isogenic control cells treated with vehicle or VPS34IN1 at 1µM for 24 hr. EV protein levels were normalized to lysate total protein (EV total) or lysate levels of the corresponding protein (EV/Lysate ratio). Bar graph denotes average protein levels normalized to control cells. (mean ± SEM, N=5-6, from two independent experiments). *p<0.05, **p<0.01, ***p<0.001 in one-way ANOVA, Holm-Sidak’s multiple comparisons test.

**
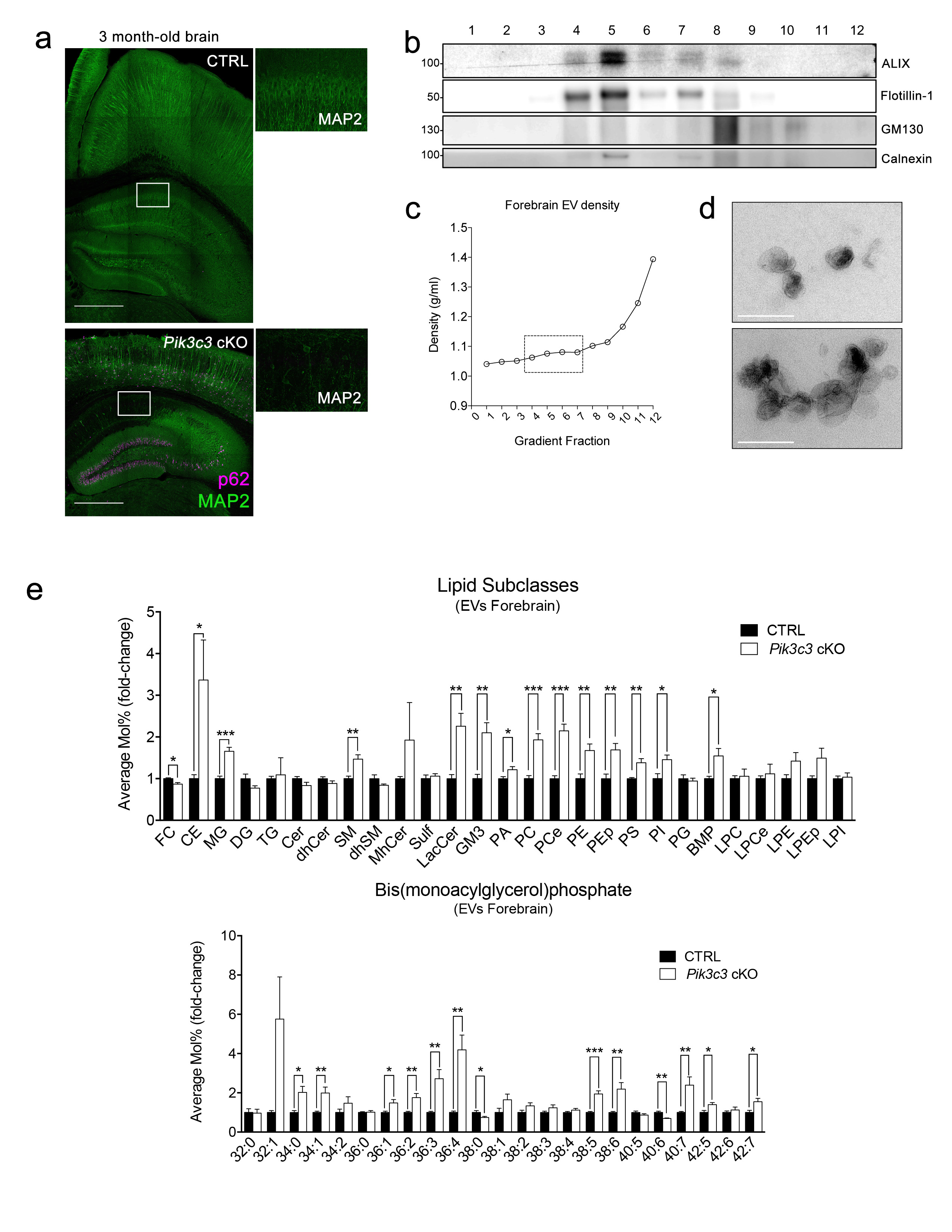
**

**Supplementary Figure 8. *In vivo* Vps34 cKO causes age-dependent neurodegeneration and secretion of EVs with a distinct lipid signature.** **a)** Brain sections from *Pik3c3*^flox/flox^ (CTRL) and *Pik3c3*^flox/flox^ *CamkIIa*-*Cre* (*Pik3c3* cKO) mice at 3 months immunostained for MAP2 and p62. Scale bar, 500µm. **b-d)** Validation of EVs purified from wild-type C57BL/6 mice and floated on an Optiprep gradient. **b)** Western blot analysis demonstrates the presence of exosomal markers ALIX and flotillin-1 in fractions 4-7. These fractions were pooled for downstream analysis. **c)** Density of recovered fractions, measured by refractometry. **d)** Representative wide-field EM images of EVs purified from wild-type C57BL/6 mice. Scale bar, 200nm. **e)** Upper panel, LC-MS analysis of lipids extracted from EVs collected from CTRL or *Pik3c3* cKO mice at 2 months. Lower panel, molecular species analysis of BMP. Values were expressed as average Mol% of total lipids measured, normalized to vehicle (mean ± SEM N=6, from two independent experiments) * p<0.05, ** p<0.01, ***P<0.01 in two-tailed Student’s t-test.

**Supplementary Figure 9. Extended data of Western Blot cropped in main and supplementary figures.** Blue rectangles indicate lanes cropped.

**Supplementary Figure 9 (continued).**

**Supplementary Figure 9 (continued).**

**Supplementary Figure 9 (continued).**

**Supplementary Figure 9 (continued).**

**Supplementary Table 1. Statistical analysis information for main and supplementary Figures.** *p<0.05, **p<0.01, ***p<0.001, color-coded by light pink, pink and red, respectively. 2t t-test: two-tailed unpaired Student’s t-test. TW RM ANOVA: two-way repeated measure ANOVA.
